# Bone marrow mesenchymal stem cells combined with Sox2 increase the functional recovery in rat with traumatic brain injury

**DOI:** 10.1186/s41016-019-0158-7

**Published:** 2019-05-15

**Authors:** Qiang Hao, Jian Zheng, Yue Hu, Hao Wang

**Affiliations:** 1 Department of Neurosurgery, Beijing Tiantan Hospital, Capital Medical University, No.119 South 4th Ring West Road, Fengtai District, Beijing, 100070 China; 2 Department of Cardiopulmonary Function Examination, Shanxi Provincial Cancer Hospital, Employee Xincun No.3, Xing Hua Ling District, Taiyuan, 030000 China; 3 Basic Medical Science Department, Capital Medical University, You An Men Wai Street, Xi Tou Tiao 10#, Fengtai District, Beijing, 100069 China

**Keywords:** Traumatic brain injury, Bone marrow stem cell, Sox2 gene, Transplantation

## Abstract

**Background:**

About 10 million individuals suffer from traumatic brain injury (TBI) each year in the world, which is one of the most serious neurological disorders. The morbidity of TBI is 55.5~6.1/100,000 in China, which takes more costing in the therapy, and the outcome of that is not well. Therefore, we expect to find new methods to treat TBI and improve the outcomes of TBI. In the previous studies reviewed, we found that stem cell transplantation may hold promising potential for modifying motor dysfunction induced by TBI.

**Methods:**

Twenty-six adult SD rats were involved in our study. Two adult SD rats were used as donors of bone marrow stem cells (BMSCs), and the other adult SD rats were divided into four groups randomly, which were used to establish the TBI models. BMSCs were transduced with lentivirous-Sox2, and we try to examine the effects of Sox2 on the differentiation of BMSCs.

**Establishment of rat TBI model:**

Rats were anesthetized using pentobarbital sodium (at a concentration of 1.5% and a dose of 40 mg/kg) and fixed under the stereotaxic device. A 1.0-cm craniotomy was performed lateral to the sagittal suture. The skullcap was carefully removed, and rats were then subjected to TBI using a controlled cortical injury instrument. A standardized parietal contusion was performed using a 20-mg steel rod with a diameter of 4 mm, which dropped from a height of 30 cm. After injury, the incision was sutured, and rats were carefully observed and nursed.

**Treatments:**

Seven days after TBI, rats were divided into four groups and were transplanted with BMSC-Sox2, single BMSC, single Lentivirus-Sox2, and PBS into injured brain, respectively. The motor function was tested using the neurological severity score (NSS).

**Results:**

We found that the ectopic expression of Sox2 enhanced BMSCs to differentiate into neurons. Seven days after TBI, the rats were treated with BMSC-Sox2, BMSC, Sox2, and PBS. Results showed that NSS were 3.352 ± 0.398 in the BMSC-Sox2 group, 4.013 ± 0.495 in the BMSC group, 4.968 ± 0.293 in the Sox2 group, and 6.257 ± 0.361 in the PBS group, suggesting that there were obvious improvements in the neurological function in BMSC-Sox2, BMSC, and Sox2 groups. In addition, the BMSC-Sox2 group had the lowest scores, *p* < 0.05.

**Conclusion:**

The ectopic expression of Sox2 could enhance BMSCs to differentiate into neurons, and intervention of BMSCs combined with Sox2 transplantation could promote recovery of motor function in rats with TBI.

## Background

About 10 million individuals suffer from traumatic brain injury (TBI) each year in the world, which is one of the most serious neurological disorders. And approximately 70,000~90,000 patients underwent long-term neurological disabilities, which will lead to great medical cost and spending more time to conduct rehabilitative care. In addition, Professor Jiang reports the morbidity of TBI is 55.5–6.1/100,000 in China, which takes more costing in the therapy, and the outcome is not well [1–4]. Unfortunately, current options for treating brain injury are limited. Therefore, to explore promising methods for treating TBI is essential and critical.

However, cell transplantation brings hope to treat TBI. As reliable cell resources, bone marrow stem cells (BMSCs) may provide great promise for regenerative medicine. Firstly, BMSCs have the ability of proliferation in vitro; secondly, they own the ability of differentiation into osteocytes, cartilages, and adipocytes [5]. In addition, it has been reported that BMSCs also have the ability of transdifferentiation into neurons and astrocytes in some reports. However, the ability of BMSCs to transdifferentiate into neurons is limited [6].

Sox2 is a transcription factor, which is highly essential in keeping the ability of self-renewal and pluripotency in embryonic stem cells (ECs) according to previous reports [7]. Sox2-positive neural stem cells (NSCs) not only proliferate as NSCs, but also differentiate into neural precursors [8].

Therefore, we study the effects of ectopic expression of Sox2 on BMSCs and explore the possibility of BMSC differentiation into neurons. In our study, we try to explore the possibility that BMSC transplantation may lead to therapeutic effects in the improvement of motor dysfunction. The goal of this study is to explore the effects of BMSC transplantation with Sox2 in a rat TBI model.

## Methods

### Experimental animals

Adult SD rats (210–250 g, 10–12 weeks old) were employed in accordance with the Capital Medical University (CMU) guidelines and were fed in the animal room under a 12-h light and dark cycle. The minimum number of rats was required according to statistical analysis. Twenty-six adult SD rats were involved in our study. Two adult SD rats were used as donors of BMSCs. The other adult SD rats were divided into four groups randomly and were used to establish the TBI models.

### Preparation BMSCs

**Fig. 1:**
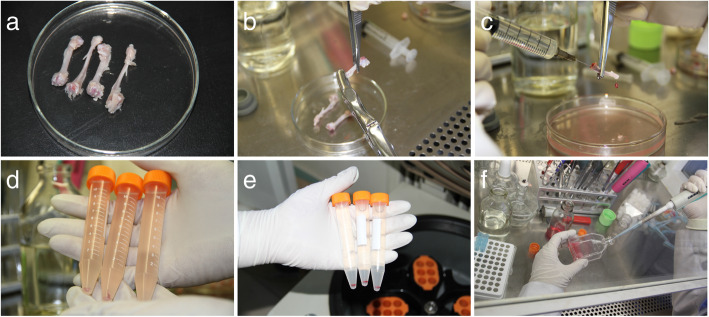
Procedure of extraction of bone marrow stem cells (BMSCs) from rat. **a** Obtaining humerus from rats. **b**, **c** Extraction of bone marrow from the humerus. **d**, **e** Bone marrow was made into a suspension and centrifuged. **f** Prepare a suspension and inoculate it in a Petri dish

BMSCs were extracted from femurs and cultured in basic culture medium (MEM, Gibco, USA), which was added into 1 mM glucose, 17% fetal bovine serum (FBS, Gibco, USA), 100 U/ml penicillin, 100 μg/ml streptomycin, and 2 mM glutamine. When cells were cultured for 24 h, the non-adherent cells were removed through changing fresh culture medium. The remaining cells were passaged and stored for future use (Fig. 1).

### Flow cytometer

BMSCs were stained with antibodies to CD34 (Abcam, Ab81289, USA) when passaged to the sixth generation and characterized by FACS analyses. After the cells reached 85% confluence, they were digested using 0.25% trypsin-EDTA and analyzed using a flow cytometer to detect the rate of CD34-positive cells.

### Differentiation of BMSCs into adipogenic and osteogenic cells

For the induction of BMSCs into adipogenic and osteogenic cells, the classical protocol was applied as previously described [5]. Briefly, 5 × 10^5^ BMSCs were seeded per well and cultured in an incubator under 37 °C with 5% CO_2_. After the BMSCs reached 100% confluence, we changed the culture medium into osteogenetic and adipogenetic differentiation medium, and cultured for additional 3 weeks. Osteogenetic differentiation medium: α-MEM, 15% FBS, 0.1 mM glumax, 0.1 mM antimycotic, 50 μg/ml l-ascorbic acid 2-phosphate, 1 nM dexamethasone, and 20 mM β-glycerolphosphate. Adipogenetic differentiation medium: α-MEM, 15% FBS, 0.1 mM glumax, 50 μM indomethacin, 0.1 mM antimycotic, 0.5 μM dexamethasone, and 0.5 μM isobutylmethylxanthine. Three weeks after induction, BMSCs were rinsed by PBS and then fixed by formalin for 1 h at RT. BMSCs for osteogenic differentiation were stained by Alizarin Red S for 20 min at RT, and cells of adipogenic differentiation were stained with oil red O for 20 min. The cells were observed using a Zeiss inverted microscope.

### Lentivirus transduction

For lentivirus infection, BMSCs were seeded at a concentration of 4 × 10^4^ cells per culture well and then were infected with Sox2-hrGFP-DU3 lentiviruses, supplemented with 0.1 g/ml Polybrene (Sigma-Aldrich) for 24 h. Lentivirus mediums were washed away using the new culture medium.

### Differentiation of BMSCs into neuron-like cells

The BMSCs were cultured in a culture medium (containing 15% FBS, 0.1 mM penicillin, 0.1 mM streptomycin, and 0.1 mM glumax) for 4 days, and then the medium was changed into neural induction culture medium, which included the neural induction medium: 50 ng/ml B27, basic FGF 50 ng/ml, FGF8 100 ng/ml, and SHH 250 ng/ml. Then, the medium was changed into neural induction medium and BMSCs were cultured for another 10 days. The neuronal induction medium was changed every 2 days.

### Immunofluorescence staining

BMSCs were cultured at a density of 500 cells per culture well and cultured on chamber slides under culture medium. Seven days later, BMSCs were fixed using a formaldehyde solution (Sigma-Aldrich, USA) at RT for 20 min. The cells were blocked with 15% normal goat serum (Vector, Burlingame, CA, USA) in PBS and coated with primary antibody anti-Tuj1 (Millipore, MA, USA) at 4 °C for 12 h. Then, the cells were incubated with goat anti-Rabbit lgG (Vector, Burlingame, CA, USA) at 1:200 dilutions for 1 h at RT. Nuclei were counterstained with DAPI (DAKO, Japan). The BMSCs were observed using a Zeiss inverted fluorescent microscope (Carl Zeiss, Germany).

### Weight-drop TBI model

Establishment of rat TBI model was performed as described by Kalish et al. with some alterations [9]. Briefly, rats were anesthetized using pentobarbital sodium (at a concentration of 1.5% and a dose of 40 mg/kg) and fixed under the stereotaxic device. A 1.0-cm craniotomy was performed lateral to the sagittal suture. The skullcap was carefully removed, and rats were then subjected to TBI using a controlled cortical injury instrument. A standardized parietal contusion was performed using a 20-mg steel rod with a diameter of 4 mm, which dropped from a height of 30 cm. After injury, the incision was sutured, and rats were carefully observed and nursed.

### Transplantation

The TBI rats were divided into four groups: BMSC transplantation group (*n* = 8), Sox2 transplantation group (*n* = 8), BMSCs-Sox2 transplantation group (*n* = 8), and sham operation group (*n* = 8). BMSCs were prepared at a density of 1 × 10^5^ cells/μl in culture medium and stored on an ice plate. When TBI rats were established for 7 days, 50 μl of BMSCs, Sox2, and BMSCs-Sox2 suspension was injected into the injured brain area using a 50-μl Hamilton syringe. The same volume of PBS was given to the sham operation group in the same way.

### Neurological function testing

**Fig. 2:**
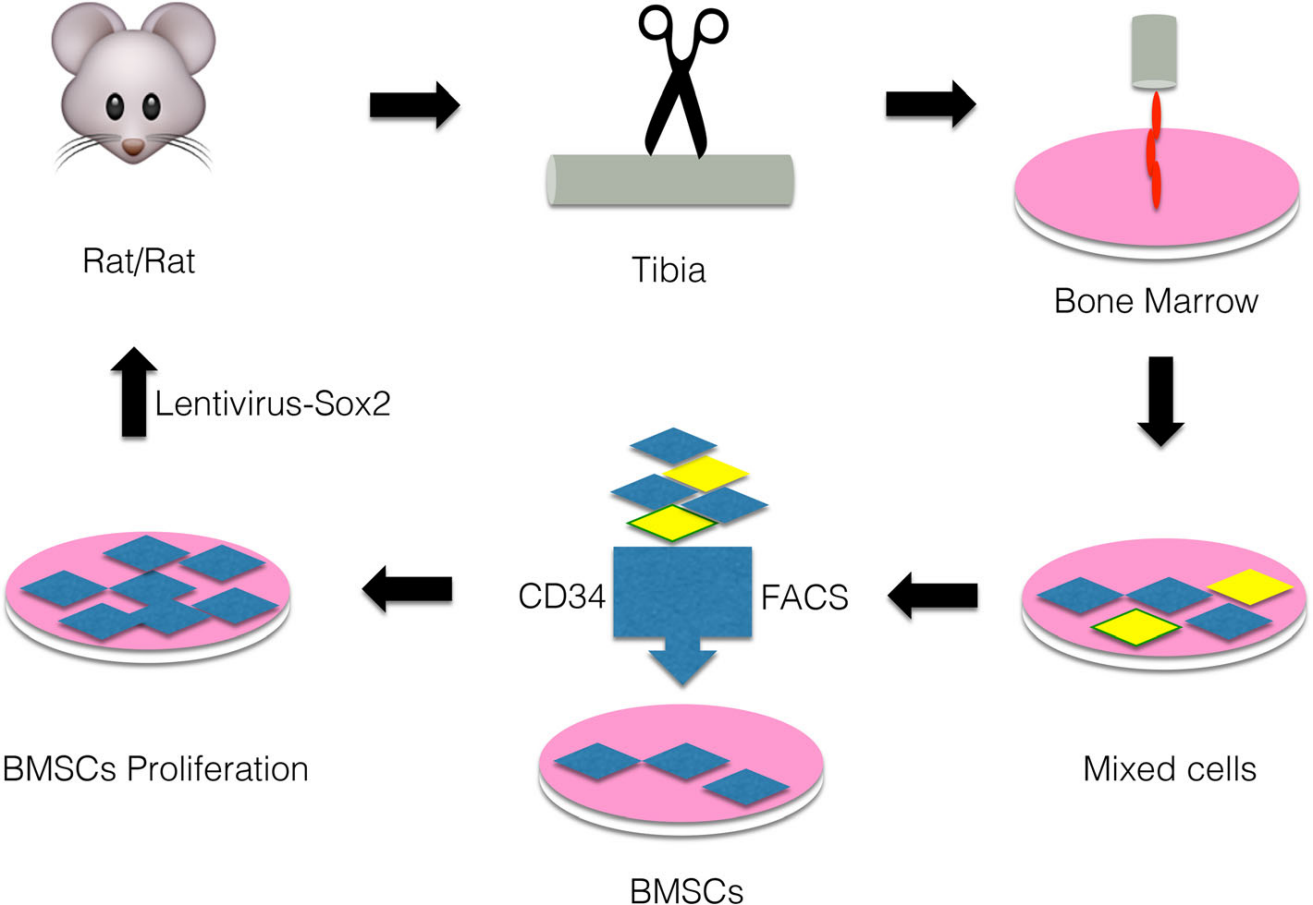
A workflow to directly indicate the process of this study

The rats were tested for motor function referring to the neurological severity score (NSS), which is described by Zhao et al., and were graded for the aspect of neurological function [10]. The relative methods are described in Fig. 6. All the rats underwent behavioral tests before TBI, 7 days after TBI, and 3 and 7 days after treatment by BMSCs, Sox2, and BMSCs-Sox2. The more severe the neurological deficit, the scores were higher. The whole procedure is described in Fig. 2.

### Statistical analysis

Statistical results of the data were analyzed by SPSS 16.0 software (USA). Data were expressed as mean ± SD. When the *p* value was less than 0.05, it was considered that there were significant differences between the different groups.

## Results

### Characteristics of BMSCs

**Fig. 3:**
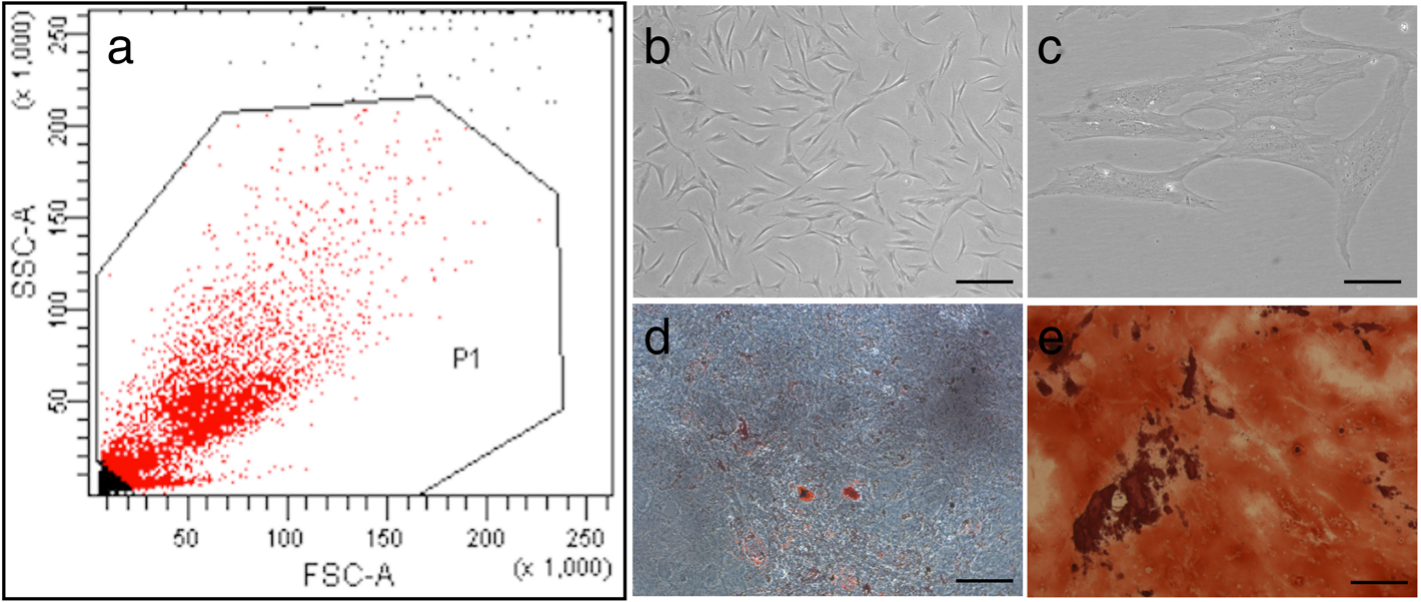
Identification of characteristics of BMSCs. **a** Selected by FACS. **b**, **c** Morphology of BMSCs. The scale bar = 50 mm. **d**, **e** Differentiation of transduced BMSCs. BMSCs were incubated in either adipogenic or osteogenic differentiation medium. The cells were fixed with 4% paraformaldehyde and stained with Oil Red O for adipogenic cells (**d**) or Alizarin Red for osteogenic cells (**e**) 3 weeks in vitro. The scale bar = 50 mm

BMSCs were selected by FACS and displayed spindle-like in the growth medium. We clarified that BMSCs had the ability of differentiation into adipocytes and osteoblasts. Results showed that Alizarin red S- and Oil red O-positive cells appeared after 3 weeks in the respective culture medium, and BMSCs cultured in the general culture medium were negative for both Oil red O and Alizarin red S staining (Fig. 3). Therefore, the BMSCs used in our study possessed the general characteristics of bone marrow stem cells.

### Ectopic expression of the Sox2 gene enhanced neuronal differentiation of BMSCs

**Fig. 4:**
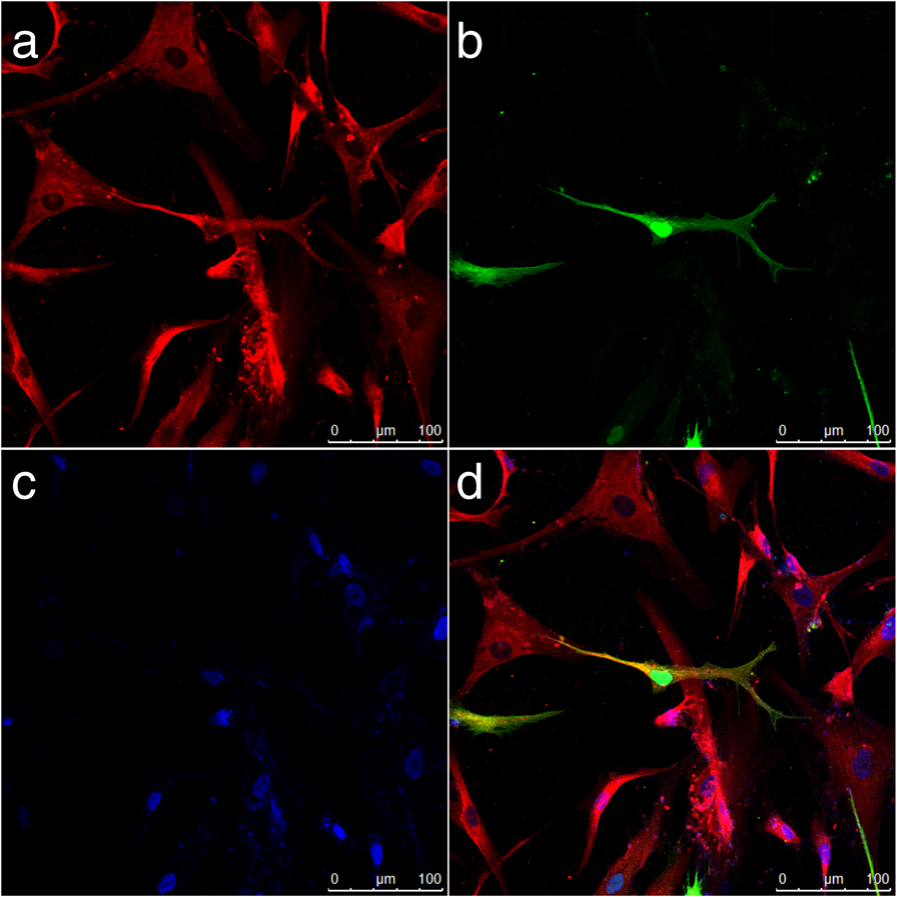
BMSC-Sox2 transdifferentiated into neurons. **a** Red is Tuj1 positive. **b** Green is rhGFP-Sox2-positive cells. **c** Blue is nuclei stained by DAPI. **d** Merge. Scale bar = 100 μm. Scale bar = 100 μm

After 7 days, the Sox2-BMSCs had large cell body, several short dendrites, and one long tubular axon located in both sides of the cell body, which were similar to common neurons. Immunohistochemistry results showed that Tuj1 was expressed in cells with axon-like processes, which were also GFP positive (Fig. 4). We speculated that the cells with long processes were neuronal-like cells. These results suggested that the ectopic expression of Sox2 could enhance BMSCs to differentiate into neurons.

### Neurological severity score

**Fig. 5:**
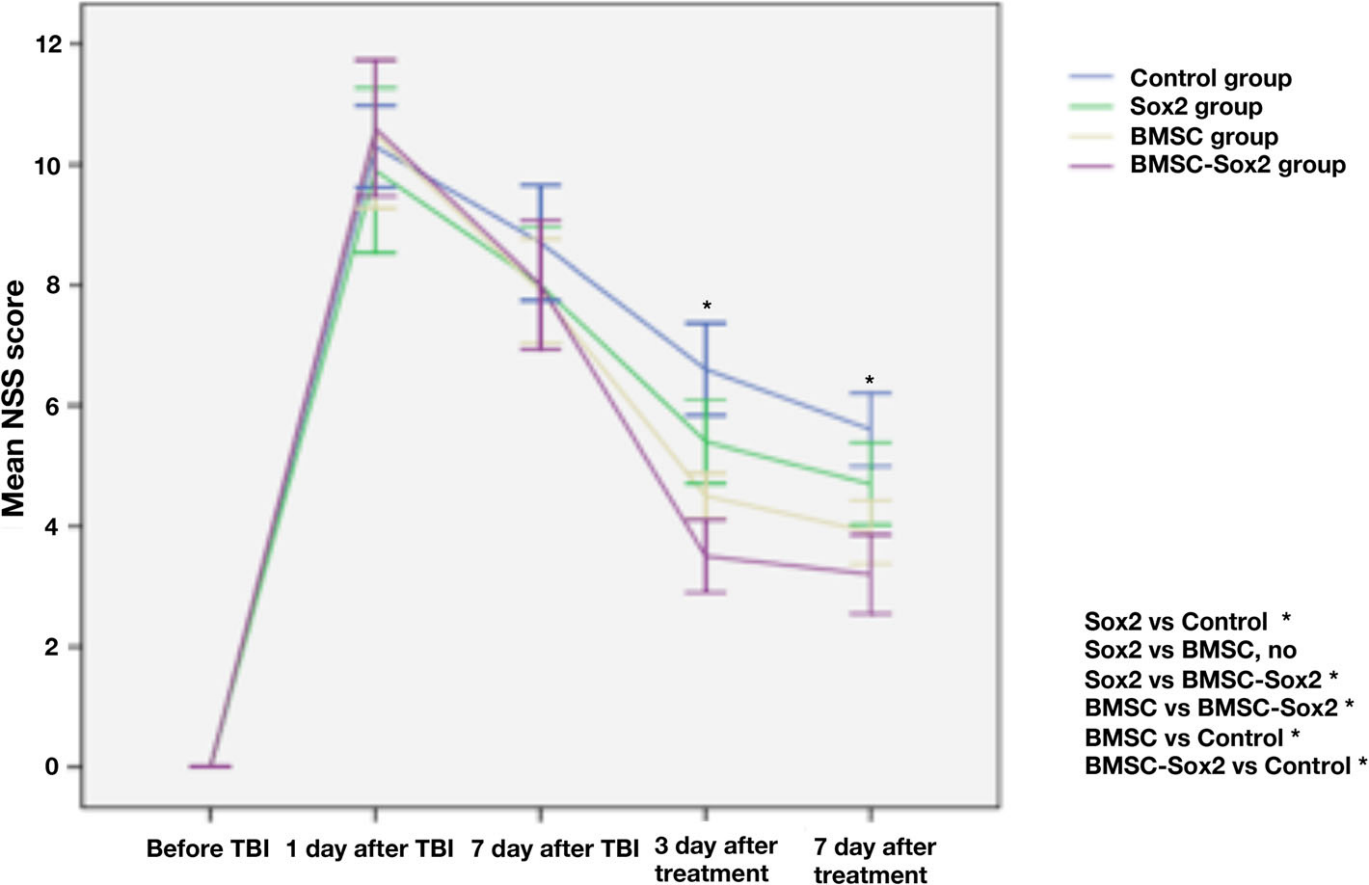
Modified neurological severity scores of rat after treatment with Sox2, BMSCs, and BMSCs-Sox2. Between the 1 and 7 days after TBI, the rats were treated with BMSC-Sox2, single BMSC, and single Lentivirus-Sox2, BMSCs-Sox2 (3.352 ± 0.398); BMSCs (4.013 ± 0.495); Sox2 (4.968 ± 0.293); Control (6.257 ± 0.361); there were significant improvements in the neurological function compared with the PBS-treated rats (*p* < 0.05; the group with BMSCs combined with Lentivirus-Sox2 gains the lowest scores (*p* < 0.05)

The TBI rats were treated with BMSC-Sox2, BMSC, Sox2, and PBS for 7 days later, and the motor function was tested using neurological severity score (NSS). Results showed that NSS were 3.352 ± 0.398 in the BMSC-Sox2 group, 4.013 ± 0.495 in BMSC group, 4.968 ± 0.293 in Sox2 group, and 6.257 ± 0.361 in the PBS group. There were significant improvements in the neurological function in the treatment group compared with the PBS group, *p* < 0.05. In addition, the BMSC-Sox2 group had the lowest scores, and there were significant differences between these groups, *p* < 0.05 (Fig. 5).

## Discussion

**Fig 6:**
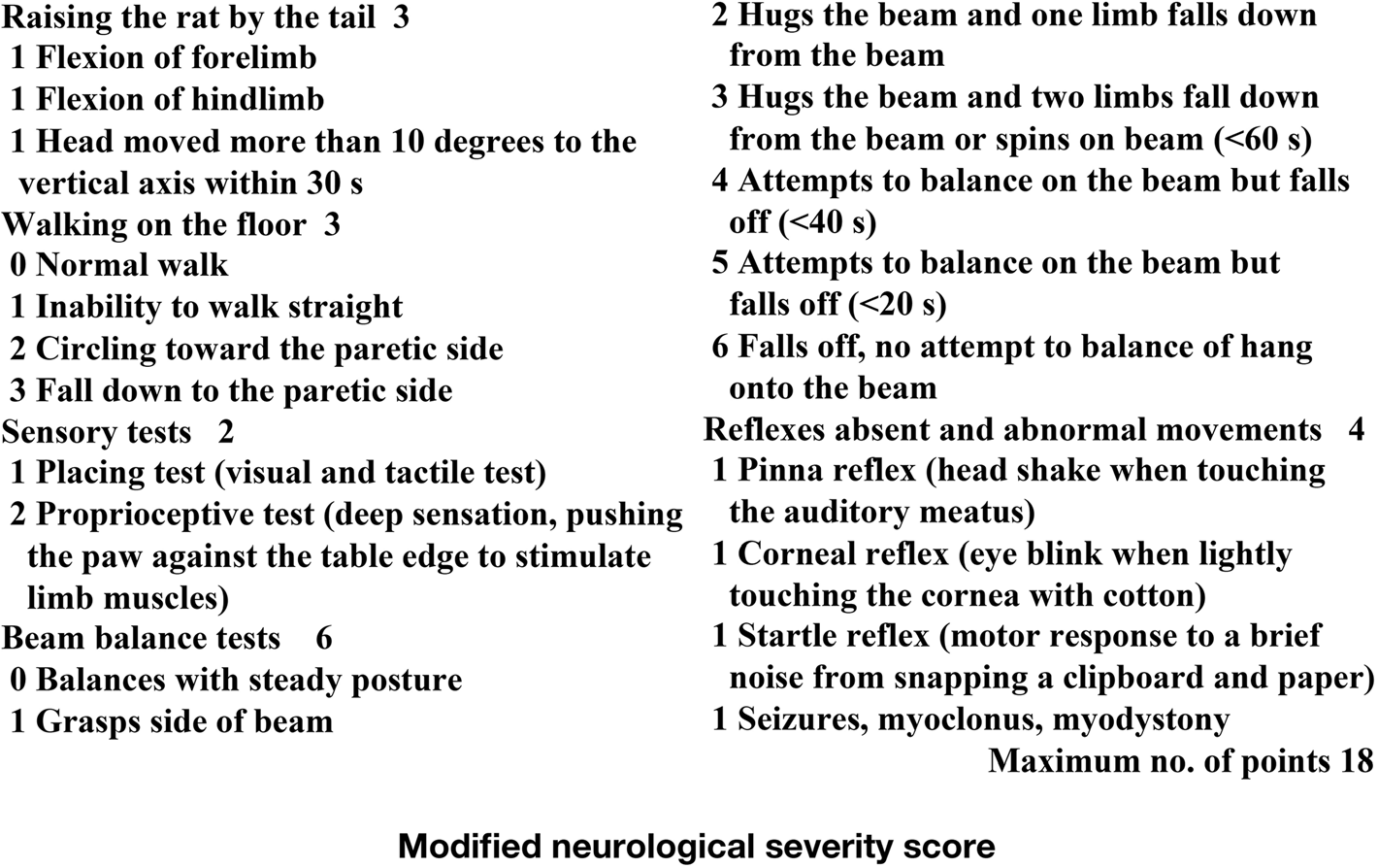
Modified neurological severity score for motor function in rats

TBI as a significant health concern takes enormous socioeconomic burden for our society. Unfortunately, to date, there are no pharmacologic agents demonstrated to improve TBI outcomes effectively. Therefore, there is a compelling need to develop treatments for TBI. The safety and feasibility of BMSC transplantation are confirmed in animal models and human trials. Our study found that cell therapies using BMSC transplantation combined with over-expression of Sox2 could improve neurological function in TBI rats, which provided basic support for the treatment of TBI (Fig. 6).

Our study was to evaluate the potential positive benefits of BMSCs with Sox2 therapy in a rat TBI model. Results showed that BMSCs with Sox2 promoted the recovery of motor function in a rat TBI model and got better outcomes. In addition, treatment of BMSCs alone and Sox2 alone also has accurate recovery of motor function, indicating that the BMSCs may provide cell sources for the re-contribution of neural function.

The adoption of BMSCs in the treatment of TBI has taken great benefits in the past years [1, 11–13]. BMSCs are not only easy to harvest but also have no immune response. More importantly, they have the ability of differentiation into cells of neuronal lineages and promote repair of neural tissues after TBI. As we have known, Sox2 functions as a core factor for stem cell pluripotency along with Oct4 and Nanog [14]. Sox2 plays a role in the development of neural progenitors in the CNS. Our data suggest that Sox2 has a role in the activation of proneural of cell fate determination.

From the above results, we can see that the recovery of nerve function requires a structural basis of functional recovery in TBI, which requires sufficient cell sources to reconstruct the damaged brain tissues. However, it is not efficient to just have a cellular basis, because of the limited ability of differentiation into neurons and other types of BMSCs. Therefore, it also needs ectopic factors to enhance the ability of differentiation into neurons and other types of BMSCs. Based on the above consideration, Sox2 owns the characteristics promoting the transdifferentiation of BMSCs into neurons and other types of cells. The results also demonstrated that in the BMSC-Sox2 group, rats with TBI have a significant improvement in the recovery of motor function.

In summary, treatment of combining BMSC and Sox2 transplantation has obtained obviously beneficial effects in neural re-contribution in a rat TBI model. However, it remains a need to explore the full effects of BMSC transplantation. More researches are required to explore the other characteristics of BMSCs for clinical applications.

## Conclusion

Sox2 can enhance the ability of differentiation of BMSCs into neurons and other cells and accelerate the recovery of motor function of rats with traumatic brain injury. BMSCs with Sox2 transplantation can promote the recovery of motor function in a rat TBI model and got better outcomes, providing support for the treatment of TBI in clinic.

## Abbreviations

BMSCs: Bone marrow stem cells
ECs: Embryonic stem cells
FBS: Fetal bovine serum
NSCs: Neural stem cells
NSS: Neurological severity score
TBI: Traumatic brain injury
